# “Just Standing Still”: A Qualitative Study on Adolescents’ Experiences of School Closures Due to Emerging COVID-19 in Bissau, Guinea-Bissau

**DOI:** 10.3390/ijerph20075265

**Published:** 2023-03-27

**Authors:** Fatou N’dure Baboudóttir, Zeca Jandi, Bucar Indjai, Jónína Einarsdóttir, Geir Gunnlaugsson

**Affiliations:** 1 Faculty of Sociology, Anthropology and Folkloristics, University of Iceland, IS-102 Reykjavik, Iceland; fnb1@hi.is (F.N.B.); je@hi.is (J.E.); 2 National Institute for Studies and Research (INEP), Avenida dos Combatentes da Liberdade da Pátria, Complexo Escolar 14 de Novembro, Bissau CP 112, Guinea-Bissau; jandizeca@gmail.com (Z.J.); indjai.b@gmail.com (B.I.)

**Keywords:** adolescents, COVID-19, school closures, education in emergencies, digital divide, qualitative research

## Abstract

The COVID-19 pandemic affected the lives of children in a myriad of ways across the world. It exposed and aggravated existing inequalities between children within countries and across continents and hampered education. In Guinea-Bissau, school closure was one of the first restrictions implemented to confront the emerging pandemic. The aim was to describe and analyse the experiences of adolescents of school closures in the capital Bissau, their concerns about their future and manifestations of inequality. Data were collected by semi-structured, open-ended interviews with 30 adolescents aged 15–17 years three months into the pandemic during an enforced state of emergency. A thematic analysis identified five themes: appreciation of education, feeling left behind, being stuck in confinement, suggestions for support, and a disrupted future. The results highlight global rather than local inequalities in the demographic, manifested by a lack of targeted educational support for public and private school students; they knew about such efforts elsewhere. The school-attending participants suggested ways to mitigate disruptions in their education, while those out of school aiming to return saw their possibilities fading away. They appreciated education for personal and national benefits, and participants worried about the long-term effects of the pandemic. The study highlighted education loss for all and disrupted future expectations.

## 1. Introduction

The COVID-19 pandemic has affected, to a varying extent, all countries across the globe since its start in late December 2019. It has resulted in disruptions in services and the daily activities of people across all layers of societies, albeit unequally [1]. Initially, children were considered to have a relatively low risk of developing severe symptoms of COVID-19 and lower case fatality rates than other age groups [2]; moreover, children were not seen as the main drivers of community transmission [3]. Nonetheless, children’s lives were affected unprecedentedly, with existing inequalities being spotlighted and exacerbated nationally and across continents [1,4,5,6,7]. 

Many governments implemented school closures early on to curb the rapid propagation of the pandemic. Rapidly, evidence was accumulated that such measures harmed children’s health and well-being, including their physical and mental health and life satisfaction [8,9]. Additionally, the school closures hampered education, while efforts in increased online teaching exposed the existing digital divide within the demographic [10,11]. In sub-Saharan Africa (SSA), with adolescents aged 10–19 years comprising 23% of the total population [12], school closures resulted in higher dropout rates, with disadvantaged students being driven into labour, and subsequent dampened future aspirations [13]. Diverse approaches were applied for remote learning in the sub-continent, including class lessons on radio, television and online platforms to avoid education loss during school closures [14]. Yet, the learning gap is estimated to grow due to the unequal access to such tools and cuts provided by education budgets [4,9,14,15,16,17]. Furthermore, school closures increased the frequency of child marriages, child pregnancies, and violence against children and resulted in worse overall health [7,8,18,19,20]. 

This research takes notice of childhood and youth studies which seek to allow children and youth to express themselves to understand the myriad of interrelationships that daily reinforce their social practice [21,22,23]. Childhood and youth studies are undergoing a theoretical and conceptual revision, creating fertile ground for critical thinking aiming at decolonising respective fields of study [24,25,26,27]. Conceptualisations of child and youth agency flourish with increased emphasis on interdependency and relational aspects [21,28], and concepts like waithood and stuckness reflect how underprivileged children and youth deal with their challenges [29,30,31,32]. Further, scholars pay increased attention to the future aspirations of the young and their emotional expressions of anticipation and hopefulness [33,34]. The commonly used binary division between children and youth into groups belonging to the Global North and Global South, respectively, risks homogenisation and a lack of differences within each group and the exaggeration of differences between groups [35,36]. Such conceptualisations are noted to be related to practices that propagate the colonisation of knowledge, namely that of the Global North deciding epistemology in terms of what kind of knowledge is of value and in what way that knowledge should be gained [37].

During the first year of the COVID-19 pandemic, most published research on children’s experiences stemmed from high-income settings, primarily relying on digital platforms [38]. As part of a decolonising effort to disseminate knowledge, we have elsewhere reported on the impact of the emerging COVID-19 pandemic in Guinea-Bissau on the lives of begging Quranic school boys [39], and how adolescents understood, and to what extent, the pandemic had affected their lives [40]. School closure was one of the governmental measures taken during the early phase of the pandemic, even before the diagnosis of the first case. Here, the aim was to describe and analyse adolescents’ lived experiences of school closure in the capital Bissau as a preventive measure in the early phase of the pandemic. Further, the aim was to hear their opinions on education, if and how school closures manifested existing inequalities within the demographic, and how the pandemic caused concerns about future aspirations.

## 2. Materials and Methods

### 2.1. Setting

Guinea-Bissau is a fragile low-income country in West Africa characterised by political instability, and it ranked 177 out of 191 on the Human Development Index 2021/2022 [41,42]. Since 2000, school enrolment has been progressing; however, the quality of education is deplorable due to the lack of school materials and inadequate teacher training [43,44,45]. Economic inequality marks access to education, and repeated teacher strikes keep public schools closed for months each year [46,47]. 

Like other countries in the West African region, Guinea-Bissau has been confronting the COVID-19 pandemic. In response to the threat, the government implemented strict restrictions on movement on 17 March 2020, while the first two cases of COVID-19 were confirmed on 25 March 2020. The government declared a state of emergency on 27 March 2020. Free movement and market activities were initially allowed between seven and eleven o´clock but gradually extended until eight o´clock in the evening as the pandemic unfolded. Further, there were restrictions on the number of people on public transport, which was initially banned but later allowed if people respected the measure of two metres of physical distancing. Furthermore, private cars were not to have more than three passengers. These restrictions were valid until 10 June 2020 and then gradually lifted. During this period, schools were closed, both public and private schools, and it was not clear when schools would be allowed to reopen. At this initial stage of the pandemic, the Bissau-Guinean population generally complied with the lockdown measures, despite its severe impact on daily lives, including those of adolescents [40]. At the time of the study, the number of confirmed COVID-19 cases was 1614 nationwide, but these were mainly in Bissau (91%), with 21 deaths being reported [48]. 

### 2.2. Research Design

Two authors (ZJ and BI) collected data on 20–24 June 2020 in 5 out of the 47 geographic areas of Bissau, the capital of Guinea-Bissau. Their selection was based on data from the national 2009 population census, but they are among the most populated ones in the capital area. Local knowledge of the setting and attention to the diverse ethnic background characterising each urban area also guided the selection. Collaborators, known to be influential people linked to associations in each neighbourhood, established contacts and permission to interview adolescents in each area. They helped to identify participants aged 15–17 years with a keen focus on gender and school attendance. In each neighbourhood, six adolescents were selected, three boys and three girls; of them, two attended public school, two attended private schools, and two had not enrolled for the current academic year. The purposive sampling aimed to maximise the diversity of participants’ experiences, not to have a representative sample [49].

The two authors interviewed 30 adolescents, giving due attention to government recommendations on COVID-19 preventive measures, such as using masks and keeping their distance. They conducted, individually, 15 interviews each, face-to-face, at a place chosen by the participants. Before the interview, they informed the participants about the study, its aims and the approximately 20–30 min interview format. The research team contextualised the semi-structured, open-ended interview guide formulated to facilitate the data collection. It was designed around eight themes, with several sub-questions per theme. The themes were: general, background, education, family, neighbourhood, friends, internet and future. The interviewers translated the guide into the participants’ lingua franca, Kriol, to enable constructive discussion, particularly on issues of concern to the respondents. It was also conducted to standardise the usage of language and the meaning of words, taking into account Kriol’s various ways of expression.

The two interviewers transcribed the audio-recorded interviews and translated them into Portuguese. In collaboration with one of the co-authors (JE), the first author (FNB) listened to all the interviews and translated the Portuguese version into English; subsequently, a content analysis of the data was performed in Atlas.ti using inductive thematic coding, also referred to as open coding [50]. In line with the grounded theory theoretical approach, the authors derived codes from coherent parts of the text rather than searching for preconceived ones. Thereafter, the codes were sorted using axial coding and merged into more extensive categories or themes [51]. After that, the key themes related to school closures and education were analysed to understand the adolescents´ experiences of school closures. 

### 2.3. Ethical Considerations

Children’s rights regarding the preventive responses to the COVID-19 pandemic include keeping them visible and listening to their voices [7,38,52]. Collaborators identified adolescents who fulfilled the study criteria in the five urban areas. Considering their mature age, they were invited to participate, and came to an agreement on a date and place for an interview with two authors (ZJ and BI). At the beginning of the interview, respondents gave their verbal consent for participation. Parental agreement was not requested, considering that the participants were 15–17 years old.

The study is one component of a larger research project that aims to articulate and explore manifestations of inequality among Bissau-Guinean adolescents in and out of school [10,39,40,46,53,54]. It was approved by the Ethics Committee of the University of Iceland (no: SHV2020-020) and by the Minister of Education in Guinea-Bissau (no/ref 250/MEES/GM/2017), in line with national regulations. 

## 3. Results

At the time of the interviews, no participant knew somebody who had become sick with COVID-19. The lockdown period was coming to an end while the schools were still closed. Five interlinked central themes emerged when considering the effect of school closures on their lives: (1) appreciation of education; (2) feeling left behind; (3) being stuck in confinement; (4) suggestions for support; and (5) a disrupted future.

### 3.1. Appreciation of Education

Although none of the questions raised inquired directly about participants’ views on the value of education, both in-school and out-of-school adolescents explained how greatly they regarded education. The out-of-school participants wanted to return to school if given the chance and considered education essential for the general development of young people. One out-of-school boy (aged 17 years) said: “When you don’t have school, what are you like: a branch, a tree without roots, because a tree without roots, … it can never develop if you don’t have school, you can never develop in society”. Others were more concerned with employment and their possibilities for future survival; for instance, an out-of-school girl (17 years) said: “I consider school important because it is the school that will help me tomorrow [in the future].” Furthermore, a few underlined that education was crucial for their country. “I don’t like to see anyone standing still,” a boy (aged 17 years) attending a public school stated, and he added “I want that we all have a good future … so that everyone can work for their country. That everyone can have an education … and be worthy citizens.”

### 3.2. Feeling Left Behind

Independent of the form of schooling, public or private, the participants were upset about the school closures. The participants who had enrolled before the school closures claimed they had not received any assistance from their schools. A girl (aged 17 years) enrolled in public school, said: “We are just standing still. The school has not found any other way to teach us.” A boy (aged 16 years) also attending public school had the same complaint: “I feel the lack of support from the school and teachers. Now, there is no support from the school to the students.”

The participants were aware that alternative online platforms were used elsewhere to provide education; however, they were deprived of such solutions due to a lack of internet access and suitable devices. A private school boy (aged 15 years) explained that “in other countries, people study through the internet, but we in Bissau do not have that, we don’t have it … lack of internet, lack of mobile, computer … we lack these things.” A girl (aged 16 years) attending a private school wanted access to online classes: “If the internet in Bissau were good, I would like the teachers to do online classes … even if they don’t teach new subjects, we could be active in those subjects that we had already received.” 

Most participants were unaware if any schools in Bissau were using alternative approaches to support their students. A public school girl (aged 17 years) mentioned she had heard about students who received some support, “but our school, they don’t give anything. If you go to our school now, you see people sitting there, but they don’t give us any tasks.” In rare cases, if someone mentioned support, it was thanks to a particular teacher. A girl (aged 16 years) in a private school gave the information that some teachers had tried to support their students through social media, but student participation was limited because of the lack of internet. Her classmates created a group on WhatsApp, but she did not participate much because she lacked internet: “I don’t know if other people have it, but my classmates created a group with the biology and ecology teacher to help us, like, if we had doubts, we send audios, to take our doubts away.” 

A few participants mentioned that they had received some support with their studies from family members. A boy (aged 17 years) who attended public school noted “my uncle sometimes takes the board out and takes the initiative to exercise the subject with us.” Another boy (aged 17 years) attending public school said “I stay at home and read books on my initiative. I had my brother who used to tutor me, but now he has stopped [helping me].”

### 3.3. Being Stuck in Confinement

The lockdown, including the school closures, changed the adolescents’ daily routines, with the participants mainly staying at home. Due to police violence, the participants and their families were obliged to remain home except for a few hours daily [40]. Due to the restrictions, most participants who had been employed within the informal sector had stopped working. Several had the same story to tell, as did a girl (aged 17 years) in public school: “I used to go to school in the morning; when I left school, I studied; when I finished studying, I would go out to sell on the road. Now I don’t do anything; I don’t sell; I only sit at home.” A few participants said they had taken up new employment and skill training during the school closures, and several mentioned that they or their friends had travelled to rural villages to help with farming activities. Nonetheless, most participants reported mainly spending their time at home during the school closures, during which some increasingly took part in family chores. Some explained that they had taken on new tasks, such as educating younger siblings and informing them about how to protect themselves from the virus. 

A few participants mentioned that thanks to the school closures, they had the opportunity to spend more time with their families and strengthened their family ties. A girl (aged 16 years) enrolled in a private school argued “we didn’t have those family ties before, but today, when there is nowhere to go, we sit, we stay having fun at home, going less out on the street.” However, most participants discussed being bored and having nothing to do except stay home during the lockdown. Many mentioned that they missed their friends and classmates or, as one participant, a girl (aged 16 years) enrolled in a private school, said, “I used to go to church and play with my classmates, but now, nothing. I just stay at home,” Likewise, a boy (aged 17 years) attending a private school complained “I practically stay at home without doing anything. I felt more comfortable when I went to school because I read subjects and did all the school tasks. But these times, I only stay at home doing nothing. That is very complicated.” Despite being stuck at home, they imagined alternatives.

### 3.4. Suggestions for Support

The participants who had enrolled before the school closures wanted support from their schools. Most of them presented ideas on how the schools could assist them in continuing their education during the lockdown; e.g., they suggested the schools could reopen with some social distancing measures in place. A girl (aged 17 years) enrolled in public school stated “I think they should reduce the number of students per class and make the use of masks compulsory; maybe that could help. Place buckets of water for handwashing at any time and avoid crowding.” A boy (15 years) enrolled in a private school argued that the school could help students and staff “to maintain hygiene in the school, to monitor the students entering the school, to bring that thermometer to see those who have that disease and those who don’t have the disease.” 

Many participants also suggested that they would have liked to have received some homework from their teachers. One girl (aged 17 years) enrolled in a public school said “they were supposed to go to school and give us some homework for us to do at home and if we finished the homework, we should bring it back to them.” Another suggested that the class leaders could act as intermediaries, bringing the homework between the teachers and the students. A girl (aged 15 years) attending a private school argued against studying at home “because we, when we stay at home, we don’t manage our time, we stay playing.” A few participants mentioned distance learning through the radio as a possibility; for instance, a girl (aged 17 years) enrolled in a private school argued “I would like the teachers to go to the radio stations to give classes. We can tune in and follow if they indicate the radio station.”

When suggesting how to continue with their studies despite the pandemic, some students mentioned that the school fees needed to be lowered to secure school access for all adolescents. A private-school boy, aged 15 years, said “there are others who can’t afford to go to school because of money. They should reduce the price of tuition and fees so that all of us can go to school.” Others pointed out that their parents or guardians had already paid the school fees and, therefore, they should be allowed to finish the school year. A private school girl (aged 17 years) said “I want them to help us finish the school year. After that, they can continue with the restrictions. Our parents paid the enrolment fees; it’s bad if we don’t finish the school year.”

### 3.5. Disrupted Future

Many participants were worried that the school closures would result in a delay in their education. “It [the pandemic] has delayed me because if we didn’t have coronavirus, I would be studying right now; we would have already taken the exams,” explained a boy (aged 15 years) enrolled in a private school. A girl (aged 17 years) attending a public school argued “we are not going to school; we are sitting here and standing still for a long time. This causes us delays because time waits for no one. Age is passing–and we are standing still”. It was nothing new for the public school students that their school was closed; before the pandemic, their schools had been annually closed for long periods because of teacher strikes. However, everything else was closed this time, with the risk of additional delay in education. A girl (aged 17 years) enrolled in a public school was worried: “I’ll have to repeat grade eight for the third time in a row, not because of successive failures, but because of the instability of the system.” Most of the out-of-school students had hoped for the possibility to enter school again; however, with the devastating economic effects of the emerging pandemic on the family economy, that dream seemed far-fetched. A participant argued that the pandemic would not change anything at all for the future; everything would return to the same conditions.

## 4. Discussion

This qualitative study aimed to understand to what extent the school closures during the state of emergency due to the unfolding COVID-19 pandemic affected the daily life and the future dreams of 15–17 years old adolescents in a low-income sub-Saharan country here in the capital Bissau, Guinea-Bissau. How does it manifest and expose existing inequalities? Key findings of the investigation include the participants’ broadly shared appreciation of education, a lack of educational support during the lockdown independent of attendance in public school vis-à-vis that in private schools, their numerous suggestions for ways of assistance and almost universal worries about disrupted futures. 

The participants, including the out-of-school ones, appreciated education’s multiple roles. They underlined its importance in securing future employment for personal development and national prosperity. While one literally praised education as a route to “worthy citizenship”, others expressed similar thoughts. Like their peers in South Africa and Uganda [13,55], the study participants in Bissau appreciated education as a route out of poverty and to a better future. Scholars have noted the high esteem attributed to education among general citizens in Guinea-Bissau, resulting in efforts to establish community schools to navigate the frequent strikes in public schools [43,56].

Independent of enrolment in private or public schools, the students lamented the lack of support for their studies during the lockdown. The COVID-19 pandemic reinforced the digital divide [11,15]; yet, for a successful implementation of digital solutions, the school, teachers, and students must be adequately equipped and prepared [57]. Although private school students in Bissau have some advantage in terms of access to digital technologies and the internet compared to public school students [10], there was, in fact, no difference between the support provided in these schools in Bissau. Occasionally, the in-school participants reported attempts to offer support which were made by an individual teacher or a group of students; in all cases, these were unsuccessful for the respective interviewee. None of the in-school participants received practical support during the lockdown from their school, which is in line with the worst scenarios [6,11,15]. In contrast, in Ghana, some schools facilitated remote learning, yet depending on family resources, students benefitted unequally [58]. The Bissau-Guinean adolescents were aware of alternative online education platforms used elsewhere. Despite the challenging circumstances, they suggested myriad ways to mitigate the consequences of the pandemic on their education. While upset about the lack of support, several participants talked in terms of rights, claiming they were entitled to support; instead, they were stuck.

Almost all participants felt they were standing still, missing their former routines of life, not least going to school. The notion of stuckness is highly contingent on the passing of time, as was reflected in a girl’s lament that she and her peers were getting older while they were “standing still.” According to Alcinda Honwana [32], the concept of waithood refers to the “prolonged period of suspension when young people’s access to social adulthood is delayed or denied.” Marjoke Oosterom [30] points out that “waithood is not about waiting or lack of engagement with the labour market, but rather about hard work, negotiation and claim making”. Before the pandemic, the study participants were busy; along with their studies, many worked within the informal sector, were engaged in house chores and enjoyed the company of peers [40]. Thus, one may argue that their everyday lives before the pandemic were characterised by active, busy waithood; in contrast, lockdown life was more like being stuck in confinement [31]. 

Almost all participants were concerned about their disrupted futures, including those out of school. Considering their general appreciation of education for individual and collective prosperity, they were upset about the expected delay in their studies, yet this was nothing new to public school students. For some, their educational level would no longer correspond to their age, while others lamented the additional delay. Out-of-school adolescents saw their dream of returning to school as being at risk of petering out. Most recognised that the pandemic posed additional threats to further education with worsened financial circumstances; thus, several participants mentioned reducing school fees as a requirement for education [6,15,59]. Ann Mische points out that “utopic end-states can be richly and lovingly imagined, while neglecting short term contingencies or alternative middle-term pathways toward realizing those futures” [60]. The eagerness of out-of-school adolescents to return to school might be realistic and rational because some manage to do so, as reflected in the high level of over-age students, particularly in public schools [46,56]. At the time of the interviews, the participants’ abilities to evaluate the short-term actions were heavily compromised, even more so than middle- or long-term ones. 

Like many other children around the globe [8], the adolescents in Bissau suffered from school closures and lost valuable time for education to secure future opportunities. Highlighting the need for education, school closure due to the COVID-19 pandemic is almost an insult to injury to the participants in an already inadequate educational system plagued by frequent teacher strikes [46,61]. School closures may have a knock-on effect if policymakers do not promptly rectify the education gap caused by the pandemic and the problems of school closure and unequal access to education [62]. Our study reveals that the prospects of young people in Bissau have been negatively impacted by the school closures caused by the pandemic, which is in line with studies from other settings worldwide [13]. However, due to a lack of educational support, access to digital technology and the internet, and other appropriate support, adolescents in Bissau were hit harder than many of their more fortunate peers elsewhere. Their voices need to be heard and acted upon to mitigate school closures’ negative impact on their future health and well-being. Further, they need to be better integrated in the development of policies concerning them and they need to be given space for active participation in decision-making processes, applying a child rights-based approach [7,38,63,64]. In the spirit of decolonising efforts, adolescents’ involvement in the co-production of knowledge on issues of concern to them could constructively contribute to the rupture of adulterish, ethnocentric and racist assumptions [25,65].

This research was conducted early in the COVID-19 pandemic in Guinea-Bissau, with lockdowns and school closures for public and private schools. Previously, school closures had been ‘reserved’ only for those attending public schools due to repeated teacher strikes. School closures became a global practice to confront the COVID pandemic. The consequences of that policy are emerging, indicating a negative impact on student achievement, particularly younger children and those of a low socioeconomic background [66]. Further, reliance on digital platforms and remote learning brings additional challenges that must be addressed for successful student learning [47,57]. In contrast to most countries, Sweden kept schools open with no reported loss in reading skills among primary school students in grades 1–3 [67]. Studies have also highlighted the negative impact of closures on children´s mental health and general well-being [8]. In settings such as Guinea-Bissau, with the frequent closures of public schools because of teacher strikes [46], globally gained experience during the pandemic, alongside our findings, should result in efforts to keep all schools open at all costs to safeguard children’s learning outcomes and their future health and well-being.

A strength of this research is that it captures the voices of adolescents on school closures in an emerging pandemic with no end in sight at the time of the study. The opinions of the enrolled students and of those out of school highlight the volatile nature of that status; all groups struggled to continue schooling and conveyed the notion of education as a route to worthy citizenship but were threatened by the school closures. It is a limitation that the sampling of participants was purposive, aiming at diversity in the adolescents’ voices regarding gender, school enrolment, habitation, and ethnicity, and it did not provide representative data. Due to the complexity of the topic, a participatory approach would have been desirable but impossible because of the general lockdown measures in place. Lastly, this study leaves out the experiences of adolescents living in urban areas outside the capital and in rural settings.

## 5. Conclusions

This research captures the diverse voices of adolescents living in the capital city of Guinea-Bissau. The data collection took place after the participants had experienced three months of a state of emergency, including the closures of all schools. Despite the generally favourable situation of adolescents enrolled in private schools compared to that of students enrolled in public ones, all the schools left their students without functioning educational support during the lockdown. Thus, rather than manifesting inequality between private vs. public school adolescents in Bissau, our study highlighted education loss for all. 

The voices of adolescents in Bissau need to be heard and acted upon to potentially mitigate some of the negative impacts the school closures have on them, including post-pandemic. Such mitigation measures must be framed within a child rights-based approach to succeed and need monitoring and further research. Of particular interest is if and how adolescents will be engaged in the recovery process, for instance, in the co-production of knowledge on matters of interest to them. Also, how much attention will be given to their worries about disrupted educational prospects and future aspirations? In the global inequality and colonial history context, will the pandemic stand out as a worldwide trigger to improve the quality and functionality of an educational system for all, or will it locally become remembered as a new, additional burden?

## Data Availability

The data presented in this study are available on request from the corresponding author. The data are not publicly available due to privacy considerations.
